# Use of Urban Residential Community Parks for Stress Management During the COVID-19 Lockdown Period in China

**DOI:** 10.3389/fpsyg.2022.816417

**Published:** 2022-03-29

**Authors:** Ni Kang, Simon Bell, Catharine Ward Thompson, Mengmeng Zheng, Ziwei Xu, Ziwen Sun

**Affiliations:** ^1^Edinburgh College of Art, University of Edinburgh, Edinburgh, United Kingdom; ^2^OPENspace Research Centre, Edinburgh College of Art, University of Edinburgh, Edinburgh, United Kingdom; ^3^Faculty of Horticulture, Chiba University, Chiba, Japan; ^4^Business School, University of Edinburgh, Edinburgh, United Kingdom; ^5^School of Design and Art, Beijing Institute of Technology, Beijing, China

**Keywords:** community park, green space, stress, lockdown, living condition

## Abstract

During the pandemic lockdown period, residents had to stay at home and increased stress and other mental health problems have been associated with the lockdown period. Since most public parks were closed, community parks within gated residential areas became the most important green space in Chinese cities, and the use of such space might help to reduce the residents’ stress levels. This study aimed to investigate to what extent urban residents in China used community parks, engaged in outdoor activity during the lockdown period (23 January–8 April 2020) and if the use of such spaces helped to reduce their stress levels. An online questionnaire survey (*n* = 1342) was carried out from 23 March to 23 April 2020. Ordinary Least Squares regression was used to analyse the association between community park use, outdoor activity, willingness to engage in outdoor activity, and stress level. All results have been further analysed by two-sampled *t*-test to explore the difference between young and old age groups. We found that the overall self-reported stress level of the respondents was relatively moderate during the lockdown period. Respondents had generally reduced their use of community parks and engagement in outdoor activity. There was no significant association between stress level and the use of community parks or the engagement in outdoor activities. However, we found that older people showed much lower stress levels, used community parks more frequently, and engaged in more outdoor activities than younger adults. The findings suggest that outdoor activities and spatial characteristics in urban China differ from Western studies and advance the need to integrate the stress management role of community parks with urban green space policy to optimise the use of community parks blended in with everyday life, particularly during the lockdown period.

## Introduction

Since the first cases were confirmed in December 2019 in China, the coronavirus infection (COVID-19) spread rapidly across the world and was declared a pandemic on 11 March 2020 by the World Health Organization [WHO] (2020a). By 23 March 2020 (the date this study survey was launched), there had been 332,930 cases confirmed globally, and the spread of the virus was continuing (World Health Organization [WHO], 2020b). The COVID-19 virus brought the risk of acute respiratory diseases and death, especially among the most vulnerable (Liu M, et al., 2020). Meanwhile, quarantine and strict lockdown measures during the pandemic have resulted in a range of psychological disorders and impact on mental health (Brooks et al., 2020; Matias et al., 2020; Torales et al., 2020). In Italy, nearly 30% of respondents to a survey reported high to extremely high degrees of depression, anxiety or stress during the lockdown period (Mazza et al., 2020), and these also occurred with cognitive failures and rumination (Lopez et al., 2021). In China, nationwide surveys launched at the early phase of the pandemic demonstrated that people have experienced anxiety, depression and stress due to the pandemic; some people also experienced severe mental disorders, especially those working in the medical field or those who knew someone infected (Ahmed et al., 2020; Cao et al., 2020; Qiu et al., 2020; Wang et al., 2020a). A survey in Japan found that compared to national psychological surveys from previous years, people were significantly more distressed even in the “mild lockdown” period (Yamamoto et al., 2020).

Extensive empirical studies have demonstrated that the use of green space (Beil and Hanes, 2013; Horiuchi et al., 2014; Lee et al., 2014; Song et al., 2015; Grazuleviciene et al., 2016; Dolling et al., 2017) and engagement in outdoor activity (Thompson, 2007; Van Den Berg and Custers, 2011; Grazuleviciene et al., 2016) have stress mitigation effects and benefits for mental health. However, in many countries, the use of green space and engagement in outdoor activities has been vastly affected by the pandemic and its associated restriction policies. For instance, studies in Scotland showed that people significantly reduced the intensity of their outdoor activity or the use of green space (Olsen and Mitchell, 2020; Stewart and Eccleston, 2020; Ugolini et al., 2020), and a similar tendency can also be found in the United States persisting beyond the end of the lockdown period (Rice et al., 2020). People also reduced the time they spent in areas further from their homes while spending more time in their local neighbourhoods (Rice et al., 2020). Furthermore, some studies reported that the greatest reduction in the use of outdoor spaces was among older people (Stewart and Eccleston, 2020; Olsen and Mitchell, 2020). Nevertheless, there have so far been only a few studies in China (e.g., Qiu et al., 2020) examining how urban residents changed their use of green space during the pandemic lockdown period.

In China, during the lockdown period (23 January to 8 April 2020), the government issued guidance on assembly limitations and measures to prevent spreading the virus but did not impose a national regulation concerning outdoor activities by ordinary uninfected people (meaning those who were not key workers) (NHCC, 2020a,b). Instead, the responsibility of controlling the movement of residents was delegated to local government and community organisations (Li et al., 2021), which led to a wide range of differences in restriction regulations from place to place. In general, urban residents in China were forced to stay at home and most amenities such as public parks were closed. Thus, community park (Supplementary Appendix Figure 1), namely, parks and garden spaces belonging to residential complexes, became the only available choice for residents if they intended to use green space.

Living areas usually consist of several residential blocks and a central, enclosed public green space (community park), all enclosed by physical boundaries, so called gated communities (Supplementary Appendix Figure 2). In the contemporary context of Chinese cities, the gated community has replaced the older publicly-owned united work-neighbourhood residential complex and has become the most common housing type in urban areas since the late 1970s (Kan et al., 2017; Guan et al., 2020). Green spaces inside a gated community are semi-public as they are normally only accessible by the community members. The particular residential type of gated community makes a significant difference between Western countries and China, as residents in Western Countries usually have private green space in their own houses, e.g., backyard or front yard gardens. While Chinese residents who live in gated communities shared a rather high density of community green space with other community members.

According to Xu and Yang (2009), residents living in such gated communities value the shared community park and use it for most of their daily outdoor activities due to features such as the high-density residential rate, the closed living environment, and the large scale of such communities. The community park turned out to be a relatively safe choice for residents if they intended to use green space (since it is semi-public) during the lockdown and was deeply integrated with residents’ everyday life. We wondered, can the community park, like other green spaces, help the residents to ease stress? So far, to our knowledge, few, if any, studies based in China have focused on how residents engaged with these community parks for their outdoor activities or for managing stress during that period. This study aimed to provide more insight into this matter.

In addition, the lockdown brought entirely different influences on people of different ages. The age difference not only occurred in COVID-19 related clinical symptoms (Liu K. et al., 2020), but also in the way people engaged in outdoor activities, which is that older people reduced them more than younger people (Mutz and Gerke, 2020). However, the situation in China differs from Western countries. As the economic reform and opening policy (from the planned to the market-oriented economy) in 1978 significantly changed Chinese society, people who grew up before the late 1970s have very different life experiences and daily life patterns from those who grew up in the new era, especially between the retired and work group (Sun et al., 2020). These might also reflect how different age groups interact differently with green space and engagement in outdoor activities in China, which needs further study in the lockdown context.

On the basis of the evidence in the literature, this study aimed to investigate the following research questions:

During the lockdown period:

(1) How did residents use community parks and engage in outdoor activity?
(2) Did being in the young and elderly age group affect the results of research questions 1?

To answer the research questions, we also hypothesised that during the lockdown period,

1. The stress level of those who participated in outdoor activities would be lower than those who did not.
2. The stress level of those who used community parks would be lower than those who did not.
3. The stress level of the older age group would be lower than that of the younger adult age group.
4. The older age group would show a more active engagement with community parks.

## Methodology

### Study Design

In order to conduct the research without face-to-face interactions (which were not possible at the time), we used the online investigation platform ‘‘SurveyStar’’ (Changsha Ranxing Science and Technology, Shanghai, China)^1^ for the pilot study and the formal online questionnaire survey. In the pilot study, seven respondents participated on 18 March 2020, followed by a telephone interview to discuss the detailed issues and to understand some unexpected answers. This helped us to further clarity the specificity of activities and spatial characteristics in the Chinese contexts and increase the questionnaire quality. For example, in theory, the community park would be the only accessible green space during the lockdown period as public parks were closed. However, the lockdown policies were different from place to place, and a couple of participants reported going out of their gated community to enjoy outside green space after the most severe period. Thus, the final version added three extra options: “drove to a suburban green space but did not get out of the car,” “walking outside the community” “exercise in outside green space/public parks.” We also added examples to explain what kinds of activities were included in each option and clearly stated where the activity was taken. In addition, we adjusted the types of living to clearly distinguish those who live in a single house from those who live in gated communities.

Subsequently, we conducted the formal questionnaire survey (detailed in “Measurements”) from 23 March to 23 April 2020, which was the last phase of the lockdown period in China. The questionnaire was shared and accessed by QR code and URL. The research team members distributed the questionnaire link widely through their social networks such as Wechat and Weibo (generally used social media software in China), encouraging the first people contacted to distribute the links further (the snowball sampling method) to reach a large number of participants. The criteria for selecting participants were: (1) age above 18 years old and (2) living in Chinese cities during the lockdown period.

The questionnaire was widely distributed resulting in participants from 98 different cities across China. After the responses were collected, we followed the data-cleaning process to check the data carefully (Van Den Broeck et al., 2005), and 1,342 valid questionnaires were collected. All the process was approved by the Edinburgh College of Art Ethics Committee (reference number: 193399-193392-56257565).

### Measurements

The questionnaire included five aspects: (1) socio-demographic data; (2) community park use during normal and lockdown periods and the reasons for using or not using the community park; (3) activities; (4) willingness to participate in outdoor activities during normal and lockdown periods (referred as WEOA); (5) stress level and perceived level of seriousness of the pandemic.

#### Socio-Demographic Data

Sociodemographic data included sex, age, occupation, residential circumstances (alone, sharing with others, family with children, and family with no children), and state of work or study (back to normal work, working from home, working from home but return to work date confirmed/not confirmed, unemployed, taking turns to work, back to school, studying at home but returning school date confirmed/not confirmed).

#### Community Park Use

Participants were first asked about their living environment (whether they had a community park); those who selected “no community park and no public space” were asked to skip the questions concerning community park use. Then, the respondents with access to a community park were asked about the frequency and duration of their use of it during lockdown and normal periods. Those who chose “never” or “only once in a few months” in the use frequency question during the lockdown period were classified as non-community-park-users. The non-community-park-users only reported what activities they had engaged in during the pandemic and the reasons constraining their use of community parks at the time. Those who responded that they had used community parks were asked one additional question concerning the reasons why they used them during the pandemic. The questions on activity, the reasons for community park use, and the constraints on community park use were all multiple-choice questions.

#### Activities

The questions on activity during the pandemic were based on discussions among the study team members who had experienced the lockdown period in China. Since the community park was only accessible for the community residents, whether lived in a gated community with a community park or not will make an impact on outdoor activities during the lockdown period. Thus, the options concerning outdoor activities were clearly stated where it was taken, such as “walking inside the community” and “walking outside the community;” “exercise inside the community” and “exercise outside the community in public spaces” (full version in Supplementary Appendix). The constraining reasons were defined based on the Leisure Barrier Model of Crawford and Godbey (1987, adjusted in 1991) (Crawford and Godbey, 1987; Crawford et al., 1991) modified to correspond to the pandemic situation by adding options such as “I obey the isolation policy” and “My family is against going out.” The Leisure Motivation Scale was used to examine the reasons for using the community park. It was initially developed by Beard and Ragheb (1980, 1983), Jim and Shan (2013), and Sreetheran (2017) further developed it by adding park visit context. We added a further option “to ease the feelings of stress/anxiety caused by the pandemic.”

#### Willingness to Engage in Outdoor Activities

The willingness to engage in outdoor activities (WEOA) during lockdown and normal period were evaluated by the participants using the Visual Analogue Scale (VAS). The VAS has frequently been used in the medical field for patients to describe their pain level (Carlsson, 1983) and its validity and reliability have also been proven in the assessment of stress (Hellhammer and Schubert, 2012), mood (Folsten and Luria, 1973; De Boer et al., 2004) and attitude (Samuelsson et al., 1997). Participants were asked to self-estimate their WEOA level using an arrow to place at a straight horizontal line providing a range of scores from 0 to 100.

#### Stress Level and Perceived Level of Seriousness of the Pandemic

Visual Analogue Scale was also applied to the measurements of stress level and perceived level of seriousness of the pandemic. We chose the VAS scale to measure perceived stress level and perceived level of seriousness of the pandemic for two reasons. First, the VAS scale is a convenient and short method, compared with other measurement tools (such as Perceived Stress Scale). According to Galesic and Bosnjak (2009). Since the quality and willingness of answering an online questionnaire declines when the length of the questionnaire increases, we intended to limit the response time to within 10 mins, and the use of the VAS could help to achieve that. Secondly, the validity of the VAS in assessing stress level has been verified in the clinical field (Lesage et al., 2012) and has also been used in testing the stress recovery effect of green space (Jiang et al., 2016).

### Data Analysis

For the initial preparation, a power analysis was applied following the instruction of Faul et al. (2009) using G^∗^Power 3.1. We used the parameters as *p* level of 0.05; medium effect size (0.25); and power of 0.90, showing a suitable sample size of 273 participants would meet the standard, and thereby, 1,342 questionnaires were enough for this study.

Descriptive statistics were first conducted to demonstrate the response percentage for sociodemographic characteristics, community park use, reasons for use/not use community park and activities during the pandemic (results in Tables 1, 2). The scores of WEOA, stress level and perceived seriousness level of the pandemic were expressed as means and standard deviations. Then OLS (Ordinary Least Squares) regression was conducted to calculate the associations between sociodemographic characteristics, community park use characteristics, WEOA, and stress level. The continuous measures like stress level, normal and pandemic WEOA were dependent variables, and sociodemographic variables (age, sex, living condition, and work) and community park related variables (living environment, community park visit frequency) were the independent variables. Table 3 presents the results of regression analysis (full version is in Supplementary Appendix). Based on the regression results, we further tested the age difference performance between younger and older groups (defined by the Chinese general retirement of age 55); living condition (with or without a companion); living environment (with or without community park) performance in WEOA, stress level, outdoor activity, community park use and reasons using two-sample *t*-test (Table 4). We tried other age group divisions (results in Supplementary Appendix), and realised that some age groups (such as those above 65) had a rather small sample size that diminished the statistical meaning of running such a model. Therefore, we devised the two age groups based on the most significant threshold of retirement age. All tests were two-tailed, with FDR (False Discovery Rate) correction of *p* value for all multiple comparison tests, significance level of ^∗^*p* < 0.05; ^∗∗^*p* < 0.01; ^∗∗∗^*p* < 0.001. All statistical analyses were undertaken in R 3.6.2 (Urbanek and Bibiko, R Foundation for Statistical Computing, 2016).

**TABLE 1:** Sociodemographic description.

Variable	Description^a^ (*N* = 1,342)
**Gender**	
Female	876 (65.28%)
Male	466 (34.72%)
**Age**	
18–30	566 (42.18%)
31–45	369 (27.50%)
46–55	245 (18.26%)
56–65	132 (9.84%)
Above 65	30 (2.24%)
**Occupation**	
Traditional industry workers	4 (0.30%)
Unemployed	32 (2.38%)
Self-employed	65 (4.84%)
Others	66 (4.92%)
Retired	92 (6.86%)
Teachers	143 (10.66%)
Private enterprise employees	238 (17.73%)
Students	343 (25.56%)
National/official employees	359 (26.75%)
**Living state during pandemic**	
Other	9 (0.67%)
Living with others	30 (2.24%)
Living in student dormitory	57 (4.25%)
Living alone	101 (7.53%)
Living in a family, without children	473 (35.25%)
Living in a family, with children	672 (50.07%)
**Household surrounding environment**	
Private garden	39 (2.91%)
With both private garden and community park	48 (3.58%)
Others	80 (5.96%)
Large scale community park	159 (11.85%)
No community park, no public space	175 (13.04%)
With public space, but no greenery	227 (16.92%)
Small-medium scale community park	614 (45.75%)
**Community management policy during pandemic**	
Others	42 (3.13%)
Complete freedom^b^	108 (8.05%)
Complete closure^c^	160 (11.92%)
Certificated residents, registered outside visitor^d^	262 (19.52%)
Certificated residents, no visitor^e^	770 (57.38%)
Normal time WEOA	60.87 (29.49)
Pandemic time WEOA	42.49 (34.48)
Perceived serious level of the pandemic	25.22 (24.85)
Pandemic stress level	34.12 (25.20)

*^a^Results are presented as frequency (%) for categorical variables and as mean (standard deviation) for continuous variables. ^b^Both outside visitors and community residents were allowed to enter/exit the community freely. ^c^Neither outside visitors nor community residents were allowed to enter/exit the community. ^d^Residents needed certification to enter/exit the community; registered outside visitors were allowed to visit. ^e^Residents needed certification to enter/exit the community; no outside visitors were allowed to visit.*

**TABLE 2:** Response of multiple choice.

	Responses^a^		Age group *t*-test		
	*N*	Percent (percent of cases)	Mean younger	Mean older	*t* value
**Activity during pandemic**					
Not answered	175				
No outdoor activity at all^b^	657	24.42% (56.30%)	0.57	0.55	−0.47
Staying alone^c^	583	21.74% (50.21%)	0.54	0.25	−6.86***
Family entertainment^d^	262	9.65% (22.50%)	0.23	0.16	−1.99*
Exercising at home	550	20.41% (47.11%)	0.46	0.53	1.46
Walking in community park	284	10.57% (24.32%)	0.21	0.45	6.67***
Exercise in community park	60	2.18% (5.14%)	0.05	0.06	0.82
Exercise in outside greenspace	76	2.82% (6.58%)	0.06	0.11	2.45**
Walk to outside greenspace	94	3.53% (8.10%)	0.07	0.14	3.06***
Drive to outside greenspace	65	2.46% (5.60%)	0.06	0.05	−0.22
Driving not getting out	28	1.00% (2.43%)	0.02	0.03	0.17
Others	33	1.20% (2.85%)	0.03	0.03	0.34
Total	2692	100% (230%)			
**Reasons for using community park during pandemic**					
Not answered	805				
Fresh air, scenery	278	23.00% (51.83%)	0.51	0.54	0.49**
Relax	262	21.74% (48.80%)	0.49	0.47	−0.40***
Ease stress	171	14.12% (31.78%)	0.32	0.30	−0.44
Sports	128	10.61% (23.82%)	0.23	0.26	0.56
Quietness	59	4.90% (11.00%)	0.11	0.12	0.36
Enjoy nature	117	9.66% (21.75%)	0.20	0.28	1.67
Accompany children	86	7.10% (16.00%)	0.19	0.05	−3.36
Accompany parents	25	2.13% (4.76%)	0.06	0.00	−2.46
Social needs	14	1.24% (2.64%)	0.03	0.01	−1.12
Pets	28	2.32% (5.20%)	0.05	0.06	0.39
Others	41	3.43% (7.55%)	0.08	0.06	−0.68
Total	1209	100% (225%)			
**Reasons for not using community park during pandemic**					
Not answered	175				
Time consuming	124	5.82% (10.66%)	0.12	0.03	−3.20***
Health concern for pandemic	555	26.00% (47.56%)	0.50	0.29	−4.94***
Isolation policy	782	36.63% (67.00%)	0.67	0.65	−0.59
Family against going outside	120	5.58% (10.31%)	0.11	0.08	−0.81
Dislike nature/outdoor	34	1.59% (2.88%)	0.03	0.00	−2.31***
Like other things better	107	5.00% (9.22%)	0.10	0.05	−2.14***
Weather concern	99	4.62% (8.46%)	0.07	0.19	5.00***
Nobody to accompany	70	3.31% (6.00%)	0.07	0.01	−2.64***
Busy with family life	66	3.14% (5.69%)	0.06	0.05	−0.27
Too crowded	63	3.03% (5.42%)	0.06	0.04	−0.89
Facility unsatisfactory	66	3.12% (5.70%)	0.06	0.01	−2.52***
Others	48	2.22% (4.12%)	0.03	0.09	3.35***
Total	2134	100% (182%)			

^a^Results are presented as frequency (%) for categorical variables and as mean (standard deviation) for continuous variables, *p < 0.05; **p < 0.01; ***p < 0.001 (with FDR correction). ^b^Except for essential reasons (shopping for food, going to hospital), did not engage with any forms of outdoor activity during the pandemic. ^c^Stayed alone and engaged in indoor entertainments that were applied alone (online charting, computer games, social media etc.). ^d^Engaged in indoor entertainments that were applied with family members (cards playing, Majiang, video games).

**TABLE 3:** Ordinary least squares (OLS) regression models linking community park use and outdoor activity to stress level (*N* = 1,342).

Variable	Stress^a^	Normal WEOA^b^	Pandemic WEOA
**Age**			
18–30	Reference	Reference	Reference
31–45	1.84 (1.68)	13.56 (1.89)***	−0.52 (2.27)
46–55	−2.58 (1.92)	18.87 (2.16)***	6.29 (2.60)*
56–65	−5.37 (2.43)	21.28 (2.73)***	16.77 (3.28)***
Above 65	−0.49 (4.71)	18.17 (5.29)***	25.81 (6.36)***
**Sex**			
Female	Reference	Reference	Reference
Male	−0.31 (1.45)	4.21 (1.69)*	4.88 (1.97)*
**Pandemic living condition**			
Living alone	Reference	Reference	Reference
Living with family, no children	−7.00 (2.75)*	−3.49 (3.22)	8.43 (3.77)*
Living with family, with children	−2.86 (2.67)	3.06 (3.13)	3.93 (3.67)
Living with others	4.05 (5.21)	1.03 (6.11)	3.77 (7.14)
Living in student dormitory	−10.08 (4.15)*	−4.07 (4.86)	−6.84 (5.69)
Others	−19.49 (8.72)*	−13.25 (10.21)	−7.02 (11.95)
**Community policy**			
Complete closure	Reference	Reference	Reference
Residents need certification, no outside visitor	0.81 (1.82)	0.45 (2.14)	1.58 (2.48)
Residents need certification, registered visitor	3.15 (2.07)	−2.05 (2.43)	2.46 (2.82)
Complete freedom	5.92 (2.77)*	−7.31 (3.25)	0.31 (3.77)
Others	−7.33 (4.09)	−13.05 (4.81)***	−4.37 (5.58)
**Living environment**			
No community park, no public space	Reference	Reference	Reference
Public space, no greenery	0.69 (2.54)	11.23 (2.89)***	8.09 (3.45)*
Medium-small scale community parks	−0.03 (2.16)	19.34 (2.46)***	11.33 (2.94)***
Large scale community parks	1.52 (2.76)	18.80 (3.15)***	11.83 (3.76)*
Private garden	−6.38 (4.47)	8.64 (5.09)	11.14 (6.08)
Both private garden and community parks	1.25 (4.11)	20.43 (4.69)***	4.40 (5.59)
Others	−0.78 (3.40)	8.81 (3.88)*	5.75 (4.63)
**Perceived seriousness of the pandemic**	0.22 (0.02)***	0.07 (0.03)	0.13 (0.04)***
**Lockdown period community park visit frequency**			
Never	Reference	Reference	Reference
Once few months	13.25 (4.74)***	−3.92 (5.32)	9.99 (6.25)
Every month	−2.26 (3.38)	0.67 (3.79)	8.33 (4.45)
Every week	2.19 (1.82)	5.66 (2.05)***	17.87 (2.40)***
Every day	1.58 (2.02)	15.69 (2.27)***	28.00 (2.66)***
**Normal period community park visit frequency**			
Never	Reference	Reference	Reference
Once few years	13.53 (12.64)	−3.88 (13.69)	10.05 (17.38)
Once a year	1.03 (12.64)	−2.13 (13.69)	11.30 (17.38)
Once few months	4.51 (3.25)	3.25 (3.52)	0.73 (4.47)
Every month	1.54 (3.54)	−1.38 (3.83)	5.20 (4.87)
Every week	3.18 (2.29)	10.97 (2.48)***	6.52 (3.15)*
Every day	1.18 (2.19)	22.85 (2.37)***	12.30 (3.01)***

^a^Results are presented as standardised regression coefficient (standard errors), *p < 0.05; **p < 0.01; ***p < 0.001 (with FDR correction). ^b^WEOA stands for “Willingness to Engage in Outdoor Activity.”

**TABLE 4:** Two sample *t*-test of different social demographic group.

Variables	Age^a^		Companion		Community park	
	Younger	Older	With	Without	With	Without
Stress	−2.20***		1.63*		0.87	
	34.66	30.15	36.69	40.92	36.66	38.00
Serious	−3.25***		2.47***		0.27	
	30.29	23.98	32.34	39.16	32.62	33.11
Normal WEOA	5.30***		0.01		−5.73***	
	59.35	71.9	63.93	63.97	67.31	57.91
Pandemic WEOA	5.95***		−0.85		−3.61***	
	40.40	57.7	49.14	46.00	51.64	44.09
Normal community park use^b^	8.20***		−1.53		−8.09***	
	14.01	20.23	14.11	12.82	11.39	15.17
Pandemic community park use	5.78***		0.13		−0.76	
	7.65	11.45	8.52	8.60	8.64	8.39

^a^Results are presented as t value on the first line, mean of each group in the second line, *p < 0.05; **p < 0.01; ***p < 0.001 (with FDR correction). ^b^Community park use score is calculated as use frequency multiply duration.

## Results

### Descriptive Result

The descriptive results of sociodemographic characteristics, WEOA during the lockdown and normal time, perceived serious level of the pandemic and the pandemic stress level are shown in Table 1. The majority of the respondents were female (65.28%) and lived with family with a child/children (50.07%). The group of participants aged 18–30 occupied the largest proportion (42.18%), followed by the 31–45 age group (27.50%). Over three-quarters of the respondents lived in a community with community parks or at least with some local public spaces. During the lockdown phase, most of the communities had applied restrictions to both residents and external visitors but extreme administrative management was not common, as situations of either complete closure or complete freedom to move only occupied 8.05 and 11.92% of situations respectively.

**FIGURE 1:**
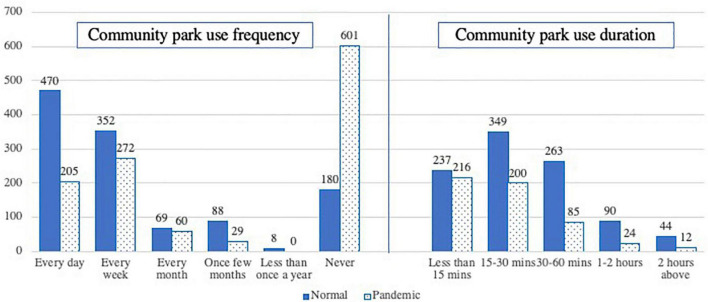
Community park use situation in normal and lockdown period (N = 1342) (Kang created).

During the lockdown period most of the respondents showed a relatively calm psychological status, as the overall mean stress level of the respondents was 34.12 ± 25.20 and the perceived seriousness level of the pandemic was 25.22 ± 24.85 (scale 0–100, higher value represents more stressful). Meanwhile, the respondents showed a large reduction in their willingness of engaging in outdoor activity during the lockdown period, as the WEOA score dropped from 60.87 ± 29.49 to 42.49 ± 34.48 (scale 0–100, higher value represents more willingness to engage in outdoor activities). As shown in Figure 1, compared with the normal period, respondents generally reduced their frequency and duration of use of community parks; and the largest proportion (44.78%) never used community parks at all during the pandemic.

The response to multiple-choice questions of outdoor activities during the lockdown period and the reasons for using/not using community parks are shown in Table 2. The internal consistency test showed that the Cronbach’s Alpha score was 0.991, 0.998, and 0.994 for each scale, indicating the high validity of the scale was used. In general, the majority of the respondents chose to stay at home and undertake no outdoor activity (24.4%) or engage in sedentary activities such as staying alone (21.7%) or entertaining together with their family (9.7%) during the pandemic. For those who used community park during the pandemic, the most frequent reasons were to enjoy the fresh air (23%), to relax (21.7%), and to ease stress (14.1%). As for the reasons for not using community parks, responding to the lock-down policy occupied the largest proportion (36.6%), followed by health concerns due to the pandemic (26%). This shows that residents’ outdoor activity and their use of community parks was significantly affected by the pandemic situation.

### Ordinary Least Squares Regression Model Result

The result of the OLS regression model which explored the association between stress level, WEOA, sociodemographic and community park use characteristics are shown in Table 3 (full version is in Supplementary Appendix). Take stress level as example, coefficients can be seen as the difference in stress level score when the independent variable is increased by on unit, with positive coefficients indicating more stressful feeling and negative coefficients indicating less stressful feeling.

#### Stress Level

As shown in Table 3, neither the use of community parks (frequency) nor the accessibly of community parks was associated with stress level during the pandemic. Some socio-demographic factors are associated with stress level. For instance, during the lockdown period, compared with those who lived alone, those who lived in a family or in student dormitories reported significantly lower stress levels, while other characteristics showed no difference on their relationship to the stress level. The reported stress level was positively associated with the perceived degree of seriousness of the COVID situation and the WEOA in the lockdown period. We tested the stress level with other socio-demographic sub-groups (e.g., under 30 years old, people who lived with accompany, etc.) and their association with stress level was also not significant (models are in Supplementary Appendix).

#### The Willingness to Engage in Outdoor Activity

Compared with the youngest age group (ages 18–30), all other age groups showed significantly more positive WEOA in the lockdown period, while in the normal period, except for the 31–45 age group, other age groups still showed significantly more positive WEOA. Male participants presenting significantly more positive WEOA than females in both periods. In the lockdown period, those who lived in a family without children reported significantly higher WEOA than those who lived alone but this tendency could not be found during the normal period. The living environment had a strong influence on WEOA, as participants who lived in environments with community parks or public spaces showed more positive WEOA compared with those who lived in environments without any greenery and public space. In both lockdown and normal period, frequent community park users (daily and weekly users) reported significant higher WEOA scores.

### Young and Elderly Group Differences

#### Differences in Stress, Willingness to Engage in Outdoor Activity, and Community Park Use

According to the OLS regression models, age difference showed a significant influence on WEOA in both the pandemic and normal periods. To investigate the age group difference further, a two-sample *t*-test with FDR correction for *p* value was applied (Table 4). The age group was divided into two categories, younger and older, the distinction is based on the general retirement age in China (55). It showed that age group difference to be significant in predicting stress level, perceived pandemic situation seriousness level, WEOA, and community park use. The younger generation felt more stressed and perceived the pandemic situation to be more serious than older participants. Meanwhile, younger participants held a significantly more negative attitude towards outdoor activity and used community parks considerably less than older participants in both pandemic and normal periods.

#### Difference in Multiple-Choice Questions

We applied the same *t*-test analysis in all multiple-choice questions to further explore the reasons for the age different performance in stress level, WEOA and community park use. To compare the results of different age groups to the total sample group, the results are integrated in Table 2. The younger generation preferred sedentary activities (staying alone and family entertainment) more than the older group, while the older group practiced significantly more outdoor activities (walking in the community park, using outside space to exercise, and walking to outside green space) than the younger group. When looking at the reasons for community park use, the younger group chose for relaxing more than the older group, while the older group chose for fresh air more than younger group, which were the only two reasons that showed age differences. In terms of reasons for not using community parks, the younger age group chose reasons such as being time-consuming, health concerns, dislike of nature, preferring to do other things to relax, lack of company and dissatisfaction with facilities more than the older group, while the older group only selected weather concerns as a reason more than the younger group.

### Other Socio Demographic Group Difference

According to the OLS regression models, other socio demographic factors also showed some differences in stress and WEOA such as the pandemic living condition and living environment. We divided the pandemic living condition as with or without companion groups (those who lived alone were seen as without companion), and the living environment as with or without community park groups (those who answered “no community park, no public space” and “with public space but no greenery” were seen as without community park group). A two-sample *t*-test with FDR correction for *p* value was applied (Table 4). It showed that those who lived with a companion during the pandemic had slightly lower stress level (*p* < 0.05) and a lower level of perceived seriousness of the pandemic. People who lived in an environment with community park showed a significantly higher level of WEOA in both normal and lockdown period. They also reported significantly more use of community park during the normal period but showed no difference during the lockdown.

## Discussion

This study investigated how residents used community parks and engaged in outdoor activity during the COVID-19 lockdown period in China in 2020. We found that the stress level of the participants was not associated with community park use and outdoor activity intensity during the lockdown period. The stress level was associated with WEOA during the lockdown period and the perceived seriousness level of the pandemic. We also found that the difference between younger and older age groups in stress level, community park use, WEOA, activities during the pandemic, and reasons for using/not using community parks was significant.

### Community Park Use

#### Stress Level and Community Park

Our result revealed that most respondents lived in an environment with community park or open space. This result is in line with the previous research, namely, a gated community with public space/community park is the mainstream living type of city dwellers in China (Guan et al., 2020), meaning that the respondents were representative of this aspect. However, the hypotheses concerning stress level has been rejected, as the use of community parks and the active engagement in outdoor activity was not associated with this. This finding is contradictory to many other studies which generally recognised the stress-reduction effect of doing outdoor activity in green space (Thompson, 2007; Van Den Berg and Custers, 2011; Grazuleviciene et al., 2016; Dolling et al., 2017), or even just viewing green space (Lee et al., 2009; Song et al., 2015). We uncovered a series of potential explanations based on the findings as follows.

First, some residents might have ignored the potential of community parks because of their daily and frequent use. A participant (female, age 30) in the pilot study mentioned that during the pandemic, her family usually drove to suburban green space to relax: *“We live in a community park environment, but we still drove to suburb parks every week to relax.”* It indicates that although people often used the community park, they might not feel it as memorable. It could mean that community park has closely integrated with residents’ everyday life, so it fails to provide novelty and attractive outdoor stimulations as public parks. Additionally, the quality and scale of community parks ranges from place to place (Miao, 2003; Xu and Yang, 2009), and these variations also influence seeing community parks as green spaces. The non-association between stress level and community park in this study did not mean that community park fell to function as green space. Contradictorily, this pointed out that community park differs from other green spaces detailed study of community park is required in the future.

Secondly, stress is a complex psychological scale that could be influenced by multiple factors, especially during the pandemic. Gao et al. (2020) found that frequent social media exposure was significantly associated with high-stress levels during the pandemic. Qiu et al. (2020) reported that the distress level of the residents was significantly associated with the availability of local medical resources, confidence in the health system, and local prevention measures. These findings suggest that the impact factors on stress levels during the lockdown were weighted in different ways. The influence of green space and outdoor activity on stress levels might be relatively small compared to other stronger factors which affected stress, and which were specific to the lockdown period. In other words, the weight change of factors on stress level could be specific to lockdown period, and the comparisons of the weight change in regular and exceptional period should be conducted in futures studies.

Moreover, our result showed that during the lockdown period, those who lived with others experienced significantly lower stress levels and perceived the seriousness of the pandemic lower than those who lived alone. The accompany of others might have played a positive and essential role on mental health during the lockdown period. As Matias et al. (2020) and Killgore et al. (2020) reported, loneliness is a noteworthy mental health concern and the need for company should be recognised during the lockdown. According to Lambert and Wang’s investigation, nearly one third of their participants reported loneliness during the lockdown period while living with a partner had a positive influence on loneliness feelings (Li and Wang, 2020). This finding also provided explanation to the non-association between stress level and community park, as we mentioned above, the stress level was influenced by other stronger factors, like companionship, rather than the use of community park. However, our finding can only provide a potential research direction in this aspect, further investigation will be required to draw a certain conclusion or in-depth discussion.

Similarly, there was no significant difference in stress level and perceived seriousness of the pandemic between people who lived with community park and those without. This supports our previous explanation that there were other factors that might have a stronger influence on stress level during pandemic than the possess of community park or the use of it. Also, we found that those who lived in environment with a community park used community parks significantly more than the opposite side in normal time, but this difference became less significant during the pandemic period. It reveals that the reduction of outdoor activity including the use of community parks during the lockdown was a universal phenomenon in various environmental characteristics.

Finally, the overall stress level of the participants might have been at a low level during the survey period (i.e., a late phase of the lockdown), when the instructions about prevention and control of the virus was relatively explicit. Studies launched at the beginning of the lockdown reported a significant mental disorders among the residents (Cao et al., 2020; Qiu et al., 2020), while a study which was launched in a late lockdown period (March 2020) reported that respondents felt satisfied with the health information and thought they were at low risk of becoming infected by COVID-19 (Wang et al., 2020b).

## Community Park and Outdoor Activity

### Two Age Groups

One of the interesting findings of this study is the effect of two age groups on stress level and engagement in green space and outdoor activity. The findings support our hypotheses concerning the stress level related to the age difference and engagement with community park use. The result indicated that the younger age group reported higher stress levels and perceived the pandemic to be more serious than the older age group, while the older age group held more positive WEOA and used community parks more than the younger group. Previous studies in China have also reported that younger groups were psychologically more vulnerable than others during the pandemic. Ahmed et al. (2020) found that people in the 21–30 age group showed significantly poorer mental well-being, higher anxiety and more depression than other groups during the pandemic (Ahmed et al., 2020). Lopez et al. (2021) also stated that as the emerging age group (between 18 and 25) was experiencing a period of changes in self-autonomy and identify, people in this age group were more sensitive to pandemic related anxious and stressful feelings. Liu M, et al. (2020) illustrated that age was negatively associated with anxiety levels during the pandemic (Liu M, et al., 2020). Higher stress levels in younger people can also be found in Japan, where Yamamoto et al. (2020) reported that people aged 18–39 suffered a significantly higher level of distress than other age groups. The negative mental health state of younger age groups during the pandemic can be understood because as the main working-age group, they needed to bear the risk of exposing themselves to work and to take the financial responsibility to support their family. Conversely, the older, retired age groups had limited financial responsibility and those who lived with their family had more opportunities to enjoy accompanying, which is beneficial to mental health.

For outdoor activities, the result showed that the younger age group engaged more in sedentary activities (staying alone, family entertainment) at home while the older group engaged more in active outdoor activities (walking, exercising, using the community park, and going out of the community). This is contrary to the Western context, where the older age group was more likely to reduce the time they spent in the outdoor environment during the lockdown period. Reports from Scotland showed that the most reduction of outdoor activity occurred among older people (although the definition of older may also be different) (Stewart and Eccleston, 2020; Olsen and Mitchell, 2020). A study in Germany also found that the younger groups remained more active outdoors than the older groups (Mutz and Gerke, 2020). A similar tendency was reported in Canada where a study showed a higher percentage of younger people engaged in outdoor activity compared with older people (Colley et al., 2020). Theoretically, the mortality of older people in COVID-19 was higher than younger people and the clinical symptoms of the older group after infection were also likely to be more serious than for the younger group (Liu K. et al., 2020). Therefore, in most Western countries, it was strongly recommended that older people should stay at home.

This difference of outdoor activity engagement between the younger and older groups in China and Western countries might have been due to various reasons, for instance, the stay-at-home policy during the pandemic. In most Western countries the guidelines clearly stated that people aged over 70 should stay at home (AG, 2021; SG, 2021), while in China the guideline did not restrict movements by age (NHCC, 2020a). As the older people in China were not warned that the threat of the virus was greater for them, they might have been more likely to go outside. Meanwhile, the specific age-based outdoor restrictions might have resulted in the significant reduction of outdoor activities among older people in Western countries. Besides this, the different types of outdoor space in Western countries and China might also contribute to the different age-related performance. Olsen and Mitchell (2020) reported that most of their respondents have private gardens, while only 2.91% of participants in our study reported having a private garden. This means that older people in Western countries might be less likely to require community green space for their exercise since they can use private gardens as substitutes. The influence of restriction policies and green space types on outdoor activity during the pandemic points to future research agendas, which could provide further explanation of the age differences in performance of younger and older groups.

Our study also revealed that older and younger age groups in China had different leisure modes. Older people were more likely to enjoy the outdoors, while younger groups preferred to stay at home doing other things for leisure. The younger groups had more reasons constraining them from using community parks, such as health concerns (pandemic-related), lack of time and preferring to do other things, than the older group. Even though the community park is an easily accessible outdoor space and the lockdown had created a long period that exempted people from work or travel, the younger people in China still showed a reluctance to use the community park or to engage in outdoor activities. The different leisure modes between young and older people not only occurred during the lockdown period. Sun et al. (2020) found that the younger group enjoyed consumption activities when going outside, while the older group preferred free amenities (e.g., public parks, city squares, community park). Moreover, instead of going outside, they might find it more relaxing to stay at home. It is not surprising that, growing up in the information era, the younger groups spent significantly more time on social media and screen use, even though studies have found a negative association between screen exposure time and mental health during the pandemic (Colley et al., 2020; Gao et al., 2020). On the other hand, the association between screen use and negative feelings might also account for the lower stress level among older people who, generally, spend less time on screen than younger groups. In addition, even in a small proportion, the younger group considered that unsatisfactory facilities was a more significant reason constraining them from using a community park than the older group. It could be that the green space was not attractive enough for them to switch their leisure activities (indoor screen using-based activities) to outdoor activities. These findings are valuable for future policymaking and the construction of community parks or public green space. Considering that younger people are more reluctant to use green space and to go outside, it is more challenging for policymakers and designers to construct green spaces attractive to the young generation.

### Limitations

The study had some limitations. First, due to the restriction of lockdown, the lack of interview data concerning why respondents showed different attitudes towards community parks and outdoor activity during the pandemic compared with normal periods. Secondly, the use of the VAS as the only analytic tool to assess stress level, WEOA, perceived seriousness of the pandemic might not be sufficient. Thirdly, the online snowballing sampling strategy might lead to the research bias caused by the under-representation of the older group and has resulted in difficulties to do comparison analysis with other age groups. Lastly, the insufficient consideration of the influence of the detailed and various community management approaches. Despite these limitations, we identified a series of future research directions, and the research team intends to improve the question setting problem in the future investigation of the post-COVID activity survey. We see this study as a foundation to explore potential research directions in the post-pandemic ear and, as in the foreseeable future the COVID-19 might coexist with human beings for a long period.

## Conclusion

This study has provided empirical evidence to answer the research questions concerning the participants’ use of community parks and engagement in outdoor activity during the pandemic and the influence of age on these aspects. We realised that the stress level during the pandemic was affected by multiple factors, and the use of green space and engagement in outdoor activity only had an indirect influence on it. Our study has also provided valuable information on community park use during the pandemic, which is, to our knowledge, innovative among the currently published studies concerning green space use. Finally, we found that the younger age group showed a higher stress level, less engagement in outdoor activities, and less use of community parks than older age groups. We believe that compared with other studies in Western countries, outdoor activities and spatial characteristics in Chinese community parks are distinct and deserve to be further studied. Such studies on the special green space (community park) can be used for the Chinese urban greenspace policy to optimise such areas in relation to residents’ everyday lives and their mental health, particularly during the lockdowns.

## Data Availability Statement

The raw data supporting the conclusions of this article will be made available by the authors, without undue reservation.

## Ethics Statement

The studies involving human participants were reviewed and approved by Edinburgh College of Art Ethics Committee (reference number: 193399-193392-56257565). Written informed consent for participation was not required for this study in accordance with the national legislation and the institutional requirements. The respondents were informed with the aims of the study and notified that the process of conducting this study has been examined by the ethical committee of Edinburgh College of Art, and if they have any questions, the contact information of the first author was provided at the beginning of the questionnaire.

## Author Contributions

NK: conceptualisation, methodology, investigation, writing—original draft, and project administration. SB: supervision, conceptualisation, and writing—review and editing. CW: supervision and conceptualisation. MZ: investigation and methodology. ZX: formal analysis and investigation. ZS: supervision, validation, and writing—review and editing. All authors contributed to the article and approved the submitted version.

## Conflict of Interest

The authors declare that the research was conducted in the absence of any commercial or financial relationships that could be construed as a potential conflict of interest.

## Publisher’s Note

All claims expressed in this article are solely those of the authors and do not necessarily represent those of their affiliated organizations, or those of the publisher, the editors and the reviewers. Any product that may be evaluated in this article, or claim that may be made by its manufacturer, is not guaranteed or endorsed by the publisher.

## Footnotes

1
https://www.wjx.cn/

## References

[B1] AG (2021). *Coronavirus a Short Guide.* Edinburgh: AgeScotland.

[B2] Ahmed M. Z. Ahmed O. Aibao Z. Hanbin S. Siyu L. Ahmad A. (2020). Epidemic of COVID-19 in China and associated Psychological Problems. *Asian J. Psychiatr.* 51:102092. 10.1016/j.ajp.2020.102092 32315963 PMC7194662

[B3] Beard J. G. Ragheb M. G. (1980). Measuring leisure satisfaction. *J. Leis. Res.* 12 20–33. 10.1080/00222216.1980.11969416

[B4] Beard J. G. Ragheb M. G. (1983). Measuring leisure motivation. *J. Leis. Res.* 15 219–228. 10.1080/00222216.1983.11969557

[B5] Beil K. Hanes D. (2013). The influence of urban natural and built environments on physiological and psychological measures of stress- A pilot study. *Int. J. Environ. Res. Public Health* 10 1250–1267. 10.3390/ijerph10041250 23531491 PMC3709315

[B6] Brooks S. K. Webster R. K. Smith L. E. Woodland L. Wessely S. Greenberg N. (2020). The psychological impact of quarantine and how to reduce it: rapid review of the evidence. *Lancet* 395 912–920. 10.1016/S0140-6736(20)30460-8 32112714 PMC7158942

[B7] Cao W. Fang Z. Hou G. Han M. Xu X. Dong J. (2020). The psychological impact of the COVID-19 epidemic on college students in China. *Psychiatry Res.* 287 1–5. 10.1016/j.psychres.2020.112934 32229390 PMC7102633

[B8] Carlsson A. M. (1983). Assessment of chronic pain. I. Aspects of the reliability and validity of the visual analogue scale. *Pain* 16 87–101. 10.1016/0304-3959(83)90088-X 6602967

[B9] Colley R. C. Bushnik T. Langlois K. (2020). Exercise and screen time during the COVID-19 pandemic. *Health Rep.* 31 3–11. 10.25318/82-003-x202000600001-eng 32672923

[B10] Crawford D. W. Godbey G. (1987). Reconceptualizing barriers to family leisure. *Leis. Sci.* 9 119–127. 10.1080/01490408709512151

[B11] Crawford D. W. Jackson E. L. Godbey G. (1991). A hierarchical model of leisure constraints. *Leis. Sci.* 13 309–320. 10.1080/01490409109513147

[B12] De Boer A. G. E. M. Van Lanschot J. J. B. Stalmeier P. F. M. Van Sandick J. W. Hulscher J. B. F. De Haes J. C. J. M. (2004). Is a single-item visual analogue scale as valid, reliable and responsive as multi-item scales in measuring quality of life? *Qual. Life Res.* 13 311–320. 10.1023/B:QURE.0000018499.64574.1f 15085903

[B13] Dolling A. Nilsson H. Lundell Y. (2017). Stress recovery in forest or handicraft environments – An intervention study. *Urban For. Urban Green.* 27 162–172. 10.1016/j.ufug.2017.07.006

[B14] Faul F. Erdfelder E. Buchner A. Lang A. G. (2009). Statistical power analyses using G*Power 3.1: tests for correlation and regression analyses. *Behav. Res. Methods* 41 1149–1160. 10.3758/BRM.41.4.1149 19897823

[B15] Folsten M. F. Luria R. (1973). Reliability, validity, and clinical application of the visual analogue mood scale. *Psychol. Med.* 3 479–486. 10.1017/S0033291700054283 4762224

[B16] Galesic M. Bosnjak M. (2009). Effects of questionnaire length on participation and indicators of response quality in a web survey. *Public Opin. Q.* 73, 349–360. 10.1093/poq/nfp031

[B17] Gao J. Zheng P. Jia Y. Chen H. Mao Y. Chen S. (2020). Mental health problems and social media exposure during COVID-19 outbreak. *PLoS One* 15:e0231924. 10.1371/journal.pone.0231924 32298385 PMC7162477

[B18] Grazuleviciene R. Vencloviene J. Kubilius R. Grizas V. Danileviciute A. Dedele A. (2016). Tracking restoration of park and urban street settings in coronary artery disease patients. *Int. J. Environ. Res. Public Health* 13:550. 10.3390/ijerph13060550 27258294 PMC4924007

[B19] Guan C. H. Srinivasan S. Zhang B. Da L. Liu J. Nielsen C. (2020). The influence of neighborhood types on active transport in China’s growing cities. *Transp. Res. D Transp. Environ.* 80:102273. 10.1016/j.trd.2020.102273

[B20] Hellhammer J. Schubert M. (2012). The physiological response to Trier Social Stress Test relates to subjective measures of stress during but not before or after the test. *Psychoneuroendocrinology* 37 119–124. 10.1016/j.psyneuen.2011.05.012 21689890

[B21] Horiuchi M. Endo J. Takayama N. Murase K. Nishiyama N. Saito H. (2014). Impact of viewing vs. Not viewing a real forest on physiological and psychological responses in the same setting. *Int. J. Environ. Res. Public Health* 11 10883–10901. 10.3390/ijerph111010883 25333924 PMC4211012

[B22] Jiang B. Li D. Larsen L. Sullivan W. C. (2016). A dose-response curve describing the relationship Between Urban tree cover density and self-reported stress recovery. *Environ. Behav.* 48 607–629. 10.1177/0013916514552321

[B23] Jim C. Y. Shan X. (2013). Socioeconomic effect on perception of urban green spaces in Guangzhou, China. *Cities* 31 123–131. 10.1016/j.cities.2012.06.017

[B24] Kan H. Y. Forsyth A. Rowe P. (2017). Redesigning China’s superblock neighbourhoods: policies, opportunities and challenges. *J. Urban Des.* 22 757–777. 10.1080/13574809.2017.1337493

[B25] Killgore W. D. S. Cloonan S. A. Taylor E. C. Dailey N. S. (2020). Loneliness: a signature mental health concern in the era of COVID-19. *Psychiatry Res.* 290:113117. 10.1016/j.psychres.2020.113117 32480121 PMC7255345

[B26] Lee J. Park B. J. Tsunetsugu Y. Kagawa T. Miyazaki Y. (2009). Restorative effects of viewing real forest landscapes, based on a comparison with urban landscapes. *Scand. J. For. Res.* 24 227–234. 10.1080/02827580902903341

[B27] Lee J. Tsunetsugu Y. Takayama N. Park B. J. Li Q. Song C. (2014). Influence of forest therapy on cardiovascular relaxation in young adults. *Evid. Based Complement. Altern. Med.* 2014:834360. 10.1155/2014/834360 24660018 PMC3934621

[B28] Lesage F. X. Berjot S. Deschamps F. (2012). Clinical stress assessment using a visual analogue scale. *Occup. Med.* 62 600–605. 10.1093/occmed/kqs140 22965867

[B29] Li L. Z. Wang S. (2020). Prevalence and predictors of general psychiatric disorders and loneliness during COVID-19 in the United Kingdom. *Psychiatry Res.* 291:113267. 10.1016/j.psychres.2020.113267 32623266 PMC7326403

[B30] Li L. Zhang S. Wang J. Yang X. Wang L. (2021). Governing public health emergencies during the coronavirus disease outbreak: lessons from four Chinese cities in the first wave. *Urban Stud.* 10.1177/00420980211049350 PMC10311377 37416836

[B31] Liu K. Chen Y. Lin R. Han K. (2020). Clinical features of COVID-19 in elderly patients: a comparison with young and middle-aged patients. *J. Infect.* 80 e14–e18. 10.1016/j.jinf.2020.03.005 32171866 PMC7102640

[B32] Liu M. Zhang H. Huang H. (2020). Media exposure to COVID-19 information, risk perception, social and geographical proximity, and self-rated anxiety in China. *BMC Public Health* 20:1649. 10.1186/s12889-020-09761-8 33148201 PMC7609828

[B33] Lopez A. Caffò A. O. Tinella L. Di Masi M. N. Bosco A. (2021). Variations in mindfulness associated with the COVID-19 outbreak: differential effects on cognitive failures, intrusive thoughts and rumination. *Appl. Psychol. Health Well Being* 13 761–780. 10.1111/aphw.12268 33765354 PMC8251010

[B34] Matias T. Dominski F. H. Marks D. F. (2020). Human needs in COVID-19 isolation. *J. Health Psychol.* 25 871–882. 10.1177/1359105320925149 32375564

[B35] Mazza C. Ricci E. Biondi S. Colasanti M. Ferracuti S. Napoli C. (2020). A nationwide survey of psychological distress among Italian people during the covid-19 pandemic: immediate psychological responses and associated factors. *Int. J. Environ. Res. Public Health* 17:3165. 10.3390/ijerph17093165 32370116 PMC7246819

[B36] Miao P. (2003). Deserted streets in a jammed town: the gated community in Chinese cities and its solution. *J. Urban Des.* 8 45–66. 10.1080/1357480032000064764

[B37] Mutz M. Gerke M. (2020). Sport and exercise in times of self-quarantine: how Germans changed their behaviour at the beginning of the Covid-19 pandemic. *Int. Rev. Sociol. Sport* 56, 305–316. 10.1177/1012690220934335

[B38] NHCC (2020a). *Prevention and Contral Plan of the COVID-19 Pandemic(Third version).* Beijing: People’s Medical Publishing House.

[B39] NHCC (2020b). *The Manual of Normalisational Prevention and Control of the COVID-19 Epidemic.* Beijing: People’s Medical Publishing House.

[B40] Olsen J. Mitchell R. (2020). *Change in Use of Green and Open Space Following COVID-19 Lockdown ‘Stay at Home’ Phase and Initial Easing of Lockdown.* Glasgow: University of Glasgow.

[B41] Qiu J. Shen B. Zhao M. Wang Z. Xie B. Xu Y. (2020). A nationwide survey of psychological distress among Chinese people in the COVID-19 epidemic: implications and policy recommendations. *Gen. Psychiatry* 33 19–21. 10.1136/gpsych-2020-100213 32215365 PMC7061893

[B42] Rice W. L. Mateer T. J. Reigner N. Newman P. Lawhon B. Taff B. D. (2020). Changes in recreational behaviors of outdoor enthusiasts during the COVID-19 pandemic: analysis across urban and rural communities. *J. Urban Ecol.* 6:juaa020. 10.1093/jue/juaa020

[B43] Samuelsson M. Sunbring Y. Winell I. Åsberg M. (1997). Nurses’ attitudes to attempted suicide patients. *Scand. J. Caring Sci.* 11 232–237. 10.1111/j.1471-6712.1997.tb00461.x 9505731

[B44] SG (2021). *Review of Physical Distancing in Scotland.* Available online at: https://www.gov.scot/publications/coronavirus-covid-19-review-physical-distancing-scotland-june-2021/pages/3/ (accessed March 21, 2021).

[B45] Song C. Ikei H. Kobayashi M. Miura T. Taue M. Kagawa T. (2015). Effect of forest walking on autonomic nervous system activity in middle-aged hypertensive individuals: a pilot study. *Int. J. Environ. Res. Public Health* 12 2687–2699. 10.3390/ijerph120302687 25739004 PMC4377926

[B46] Sreetheran M. (2017). Exploring the urban park use, preference and behaviours among the residents of Kuala Lumpur, Malaysia. *Urban For. Urban Green.* 25 85–93. 10.1016/j.ufug.2017.05.003

[B47] Stewart D. Eccleston J. (2020). *Enjoying the Outdoors: Monitoring the Impact of Coronavirus and Social Distancing. NatureScot Research Report No. RR1255.* Inverness: NatureScot.

[B48] Sun Z. Lai K. Y. Bell S. Scott I. Zhang X. (2020). Exploring the associations of walking behavior with neighborhood environments by different life stages: a cross-sectional study in a smaller Chinese city. *Int. J. Environ. Res. Public Health* 17:237. 10.3390/ijerph17010237 31905693 PMC6982100

[B49] Thompson C. W. (2007). Adult Visits to Green Places. *Environ. Behav.* 40 111–143.

[B50] Torales J. O’Higgins M. Castaldelli-Maia J. M. Ventriglio A. (2020). The outbreak of COVID-19 coronavirus and its impact on global mental health. *Int. J. Soc. Psychiatry* 66 317–320. 10.1177/0020764020915212 32233719

[B51] Ugolini F. Massetti L. Calaza-Martínez P. Cariñanos P. Dobbs C. Ostoic S. K. (2020). Effects of the COVID-19 pandemic on the use and perceptions of urban green space: an international exploratory study. *Urban For. Urban Green.* 56:126888. 10.1016/j.ufug.2020.126888 33100944 PMC7566824

[B52] Van Den Berg A. E. Custers M. H. G. (2011). Gardening promotes neuroendocrine and affective restoration from stress. *J. Health Psychol.* 16 3–11. 10.1177/1359105310365577 20522508

[B53] Van Den Broeck J. Cunningham S. A. Eeckels R. Herbst K. (2005). Data cleaning: detecting, diagnosing, and editing data abnormalities. *PLoS Med.* 2:e267. 10.1371/journal.pmed.0020267 16138788 PMC1198040

[B54] Wang C. Pan R. Wan X. Tan Y. Xu L. Ho C. S. (2020a). Immediate psychological responses and associated factors during the initial stage of the 2019 coronavirus disease (COVID-19) epidemic among the general population in China. *Int. J. Environ. Res. Public Health* 17:1729. 10.3390/ijerph17051729 32155789 PMC7084952

[B55] Wang C. Pan R. Wan X. Tan Y. Xu L. McIntyre R. S. (2020b). A longitudinal study on the mental health of general population during the COVID-19 epidemic in China. *Brain Behav. Immun.* 87 40–48. 10.1016/j.bbi.2020.04.028 32298802 PMC7153528

[B56] World Health Organization [WHO] (2020a). *Coronavirus Disease 2019 (COVID-19) Situation Report –23.03.2020.* Available online at: https://www.who.int/emergencies/diseases/novel-coronavirus-2019 (accessed March 21, 2021).

[B57] World Health Organization [WHO] (2020b). *WHO Director-General’s Opening Remarks at the Media Briefing on COVID-19 - 11 March 2020. WHO Dir. Gen. speeches*, 4. Available online at: https://www.who.int/dg/speeches/detail/who-director-general-s-opening-remarks-at-the-media-briefing-on-covid-19—11-march-2020 (accessed March 21, 2021).

[B58] Xu M. Yang Z. (2009). Design history of China’s gated cities and neighbourhoods: prototype and evolution. *Urban Des. Int.* 14 99–117. 10.1057/udi.2009.12

[B59] Yamamoto T. Uchiumi C. Suzuki N. Yoshimoto J. Murillo-Rodriguez E. (2020). The psychological impact of “mild lockdown” in Japan during the COVID-19 pandemic: a nationwide survey under a declared state of emergency. *Int. J. Environ. Res. Public Health* 17:9382. 10.3390/ijerph17249382 33333893 PMC7765307

