# Supplementary material for: Use of Urban Residential Community Parks for Stress Management During the COVID-19 Lockdown Period in China
Source: Front Psychol. 2022 Mar 29;13:816417. doi: 10.3389/fpsyg.2022.816417 (PMC9001956; doi:10.3389/fpsyg.2022.816417)
Supplement: Supplementary file 1 [file Data_Sheet_1.pdf]

Appendix

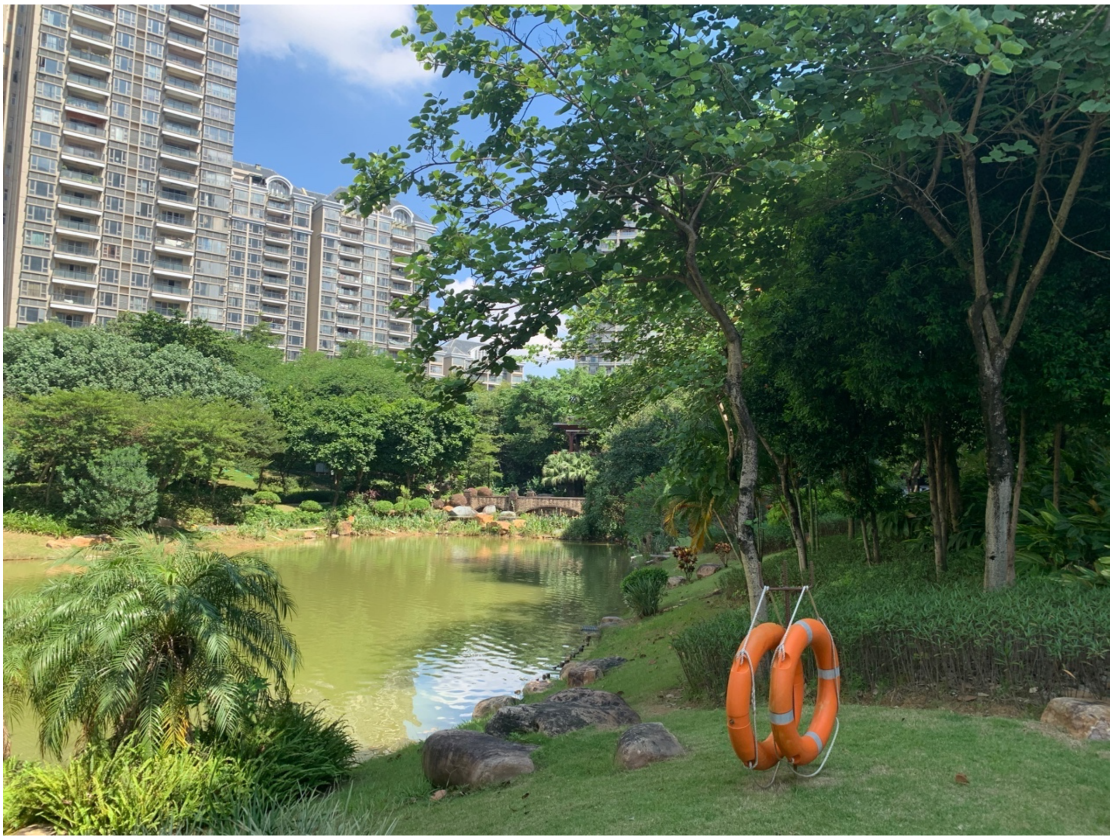

Supplementary Figure. 1 Sample image for community park (photo took on 27/05/2021 by Kang with location information concealed)

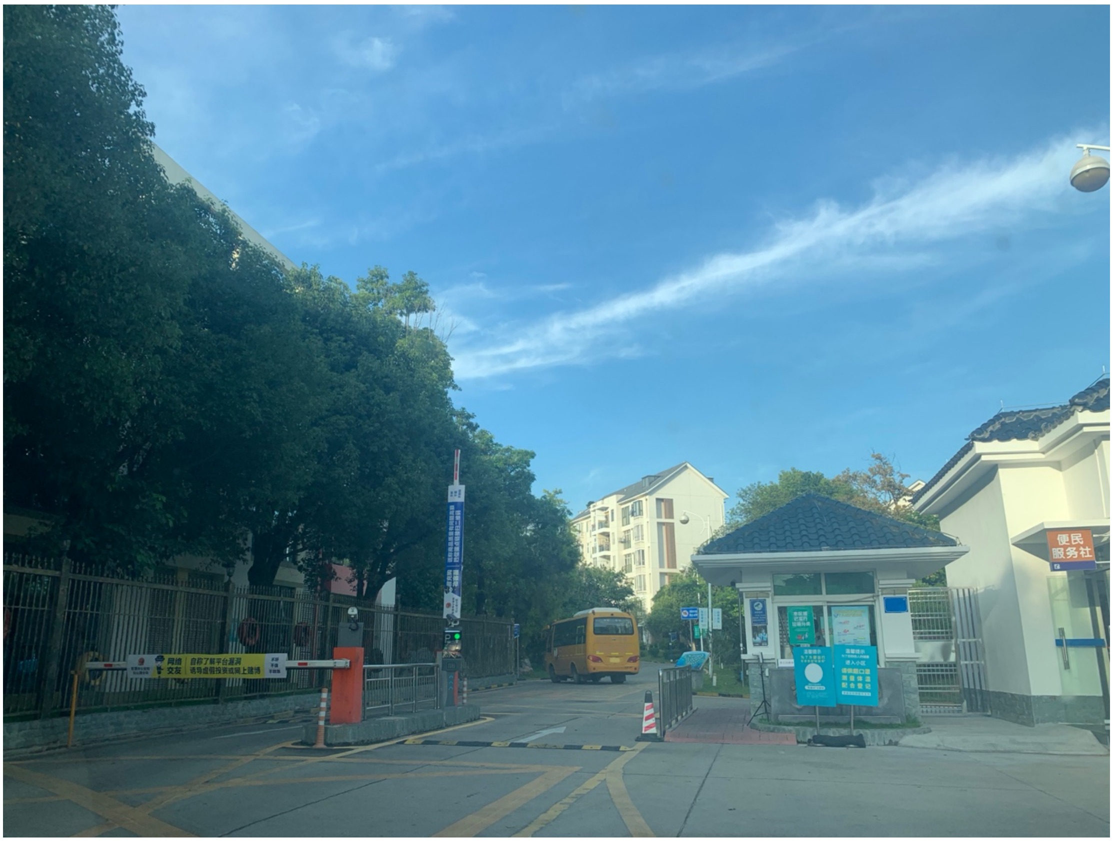

Supplementary Figure. 2 Sample image for gated community (photo took on 27/05/2021 by Kang with location information concealed)

Questions that concerning of the particular living type (gated community) in China[a.]
7. During the lockdown period, the management policy of your residential area is?
Complete closure
Limited times of leaving/entering the community, no visitors
Residents need certification to leave/entre the community (no limits on the number of access), no visitors
Residents need certification to leave/entre the community, visitors allowed (with registration)
Complete free (no limitation or registration requirement for both visitors and residents)
Others
8. Which of the following categories does the environment of your current home fall into?
No community park, no public space (single house)
With public space but no community park (no greenery)
With small-medium size community park and with some facilities in the community
With large size community park and abundant facilities in the community
With private garden/space
With both private garden and community park
Others
10. During the lockdown period, what are your entertainment activity and outdoor activity (except for work needs)? (multiple choice)
Except for living needs (like shopping for food, bringing up take-outs, etc.), I engaged in no outdoor activity
Staying alone (e.g. reading, gaming, online chatting)
Family entertainment (e.g. card games, Majiang, chatting)
Exercise at home
Taking a walk in the community
Exercise inside the community (e.g. dancing, running, basketball, fishing)
Walking outside the community (outside green space, public parks)
Exercise in outside green space/public parks
Driving to outside space to relax, but not getting off the car
Others

a. In the Chinese context, the word “community” (in Chinese “Xiaoqu”) refers to “gated community”,

Supplementary Table 1. Full version of OLS regression models (N=1342)[a.]

Variable	Stress	Normal WEOA[b.]	Pandemic WEOA	Activity[c.]
**Age**				
18-30	Reference	Reference	Reference	Reference
31-45	1.84(1.68)	13.56(1.89)***	-0.52(2.27)	-0.69(0.23)***
46-55	-2.58(1.92)	18.87(2.16)***	6.29(2.60)*	-0.31(0.26)
56-65	-5.37(2.43)	21.28(2.73)***	16.77(3.28)***	0.72(0.33)*
Above65	-0.49(4.71)	18.17(5.29)***	25.81(6.36)***	0.33(0.62)
**Sex**				
Female	Reference	Reference	Reference	Reference
Male	-0.31(1.45)	4.21(1.69)*	4.88(1.97)*	0.19(0.20)
**Occupation**				
Traditional industry	Reference	Reference	Reference	Reference
Employee of private enterprises	-2.39(12.7)	-27.46(14.4)	-25.53(17.27)	0.21(1.62)
Inoccupation	7.03(13.35)	-22.97(15.14)	-18.28(18.16)	0.3(1.71)
National/official employee	1.33(12.66)	-19.55(14.36)	-20.36(17.22)	0.55(1.62)
Others	1.36(12.97)	-24.45(14.7)	-23.02(17.64)	-0.53(1.66)
Retired	-5.22(12.86)	-14.82(14.58)	-8.49(17.49)	0.97(1.65)
Self-employment	0.55(12.97)	-23.4(14.71)	-20.1(17.64)	0.15(1.67)
Student	0.59(12.66)	-36.54(14.36)**	-24.03(17.22)	1.37(1.63)
Teacher	1.21(12.77)	-16.83(14.47)	-14.33(17.36)	1.18(1.63)

Supplementary Table 1. (Continued)

Variable	Stress	Normal WEOA	Pandemic WEOA	Activity
**Study/work state**				
Back to work	Reference	Reference	Reference	Reference
Online study, resuming time not confirm	-0.25(1.63)	-8.85(1.89)***	0.82(2.23)	0.88(0.23)***
Others	1.84(2.28)	1.2(2.65)	7.82(3.12)	0.03(0.300)
Work from home, resuming time not confirm	4.31(3.87)	4.41(4.52)	12.90(5.28)*	0.53(0.55)
Take turns to work	2.89(4.51)	2.03(5.25)	-6.05(6.17)	0.41(0.62)
Not working	6.19(4.51)	-3.00(5.25)	-2.62(6.17)	0.44(0.62)
Back to school	-5.64(4.64)	-3.92(5.4)	3.96(6.35)	-0.27(0.67)
Taking no course	3.84(4.64)	-14.43(5.4)***	-10.01(6.35)	-0.44(0.65)
Work from home, resuming time confirmed	8.65(5.59)	6.39(6.51)	-1.60(7.65)	0.54(0.78)
**Pandemic Living condition**				
Living alone	Reference	Reference	Reference	Reference
Living with family, no kids	-7.00(2.75)*	-3.49(3.22)	8.43(3.77)*	0.81(0.40)**
Living with family, with kids	-2.86(2.67)	3.06(3.13)	3.93(3.67)	0.25(0.38)
Living with others	4.05(5.21)	1.03(6.11)	3.77(7.14)	0.00(0.74)
Living in student dormitory	-10.08(4.15)*	-4.07(4.86)	-6.84(5.69)	0.23(0.57)
Others	-19.49(8.72)*	-13.25(10.21)	-7.02(11.95)	0.98(1.20)
**Community policy**				
Complete closure	Reference	Reference	Reference	Reference
Residents need certification, no outside visitor	0.81(1.82)	0.45(2.14)	1.58(2.48)	0.68(0.25)***
Residents need certification, registered visitor	3.15(2.07)	-2.05(2.43)	2.46(2.82)	0.87(0.29)*
Complete freedom	5.92(2.77)*	-7.31(3.25)	0.31(3.77)	1.29(0.45)*
Others	-7.33(4.09)	-13.05(4.81)***	-4.37(5.58)	0.07(0.60)

Supplementary Table 1. (Continued)

Variable	Stress	Normal WEOA	Pandemic WEOA	Activity
**Living environment**				
No community park, no public space	Reference	Reference	Reference	Reference
Public space, no greenery	0.69(2.54)	11.23(2.89)***	8.09(3.45)*	-
Medium-small scale community parks	-0.03(2.16)	19.34(2.46)***	11.33(2.94)***	0.26(0.25)
Large scale community parks	1.52(2.76)	18.8(3.15)***	11.83(3.76)*	0.17(0.34)
Private garden	-6.38(4.47)	8.64(5.09)	11.14(6.08)	-0.18(0.56)
Both private garden and community park	1.25(4.11)	20.43(4.69)***	4.4(5.59)	0.12(0.52)
Others	-0.78(3.4)	8.81(3.88)*	5.75(4.63)	-0.87(0.42)*
Perceived seriousness of the pandemic	0.22(0.02)***	0.07(0.03)	0.13(0.04)***	-0.00(0.00)
**Lockdown period community park visit frequency**				
Never	Reference	Reference	Reference	Reference
Once few months	13.25(4.74)***	-3.92(5.32)	9.99(6.25)	1.26(0.59)*
Every month	-2.26(3.38)	0.67(3.79)	8.33(4.45)	1.16(0.40)***
Every week	2.19(1.82)	5.66(2.05)***	17.87(2.40)***	2.1(0.23)***
Everyday	1.58(2.02)	15.69(2.27)***	28(2.66)***	1.97(0.25)***
**Normal period community park visit frequency**				
Never	Reference	Reference	Reference	Reference
Once few years	13.53(12.64)	-3.88(13.69)	10.05(17.38)	-0.71(1.62)
Once a year	1.03(12.64)	-2.13(13.69)	11.3(17.38)	1.29(1.62)
Once few months	4.51(3.25)	3.25(3.52)	0.73(4.47)	0.35(0.43)
Every month	1.54(3.54)	-1.38(3.83)	5.2(4.87)	0.42(0.46)
Every week	3.18(2.29)	10.97(2.48)***	6.52(3.15)*	1.37(0.29)***
Everyday	1.18(2.19)	22.85(2.37)***	12.3(3.01)***	1.28(0.28)***

a. Results are presented as standardised regression coefficient (standard error), *$p<0.05$; **$p<0.01$; ***$p<0.001$.
b. WEOA stands for "Willingness to Engage in Outdoor Activity"
c. Activity stands for "Activity intensity level", the calculation and weighting is in Supplementary Table 2.

Supplementary Table 2. Weighting for outdoor activity

Activity	Weight	Activity type
Nearly no outdoor activity (except for necessary shopping and work)	1	Sedentary activity
Staying alone at home		
Family entertainment		
Exercising at home	2	Light activity
Driving but no getting out		
Walking to outside greenspace	3	Light-moderate activity
Walking in community park		
Use community park to exercise	4	Moderate-vigorous activity
Use outside space to exercise		
Drive to outside greenspace		

Supplementary Table 3. OLS regression model of stress level in different socio-demographic sub-groups[a.]

Variable	Under 30	Over 30	Under 45	Over 30	With Company	With community park
**Age**						
18-30	Reference	Reference	Reference	Reference	Reference	Reference
31-45	-	-	-	-	0.958(1.912)	2.271(2.095)
46-55	-	-	-	-	-2.239(2.168)	-2.048(2.321)
56-65	-	-	-	-	-4.259(2.739)	-3.428(2.864)
above 65	-	-	-	-	-0.121(5.064)	3.868(5.171)
**Gender**						
Female	Reference	Reference	Reference	Reference	Reference	Reference
Male	-2.018(2.715)	-0.459(1.920)	-0.262(1.955)	-1.582(2.608)	-1.065(1.617)	-0.835(1.733)
**Community Policy**						
Complete closure	Reference	Reference	Reference	Reference	Reference	Reference
Residents need certification, no outside visitor	-2.632(4.313)	0.182(2.891)	-1.072(3.138)	0.892(3.676)	0.0474(2.508)	-0.830(2.572)
Residents need certification, registered visitor	0.0312(4.501)	1.347(3.353)	1.018(3.399)	0.988(4.301)	2.426(2.775)	2.024(2.935)
Complete freedom	3.161(5.272)	-0.219(6.270)	2.575(4.425)	5.549(8.234)	4.378(3.883)	7.157(4.468)
**Living Environment**						
No community park, no public space	Reference	Reference	Reference	Reference	Reference	Reference
Public space, no greenery	4.468(4.952)	1.561(4.778)	2.459(3.836)	1.239(7.596)	2.623(3.450)	-
Medium-small scale community parks	6.411(4.770)	-0.479(4.316)	3.099(3.610)	-2.820(7.008)	2.219(3.218)	2.674(3.190)

Supplementary Table 3. (continued)

Variable	Under 30	Over 30	Under 45	Over 30	With Company	With community park
Large scale community parks	1.468(5.661)	4.022(4.804)	4.708(4.198)	-2.464(7.537)	2.898(3.678)	4.139(3.625)
Private garden	-2.583(7.579)	-5.503(6.539)	-6.455(6.109)	-5.765(8.920)	-6.225(5.162)	-4.381(4.914)
Both private garden and community parks	-8.430(8.364)	7.025(5.932)	-2.018(5.781)	10.23(8.774)	3.431(4.733)	4.088(4.704)
**Perceived Seriousness**	0.199***(0.0479)	0.226***(0.0399)	0.171***(0.0360)	0.337***(0.0574)	0.211***(0.0318)	0.206***(0.0340)
**Normal period community park visit frequency**						
Never	Reference	Reference	Reference	Reference	Reference	Reference
Everyday	1.529(3.948)	1.347(3.153)	4.838(2.881)	-5.659(4.441)	2.260(2.485)	-0.0126(2.734)
Every week	4.395(3.557)	2.157(3.406)	3.333(2.820)	-0.933(4.860)	2.938(2.521)	1.172(2.788)
Every month	3.164(4.949)	2.755(5.293)	5.835(4.121)	-9.792(7.287)	4.236(3.644)	3.538(4.138)
once few months	4.731(4.588)	-1.865(4.918)	4.382(3.765)	-8.426(7.336)	2.083(3.428)	0.187(3.888)
once a year	-8.159(14.51)	10.47(24.81)	-1.388(12.66)		-2.654(12.42)	5.211(14.39)
once few years	20.77(18.36)	5.202(17.95)	14.21(14.84)	16.77(23.99)	10.72(12.57)	3.865(17.54)
**Pandemic period community park visit frequency**						
Never	Reference	Reference	Reference	Reference	Reference	Reference
Everyday	6.794(4.153)	2.627(2.588)	1.797(2.980)	6.259(3.259)	3.757(2.240)	2.154(2.353)
Every week	1.587(3.244)	1.326(2.445)	1.313(2.415)	3.103(3.252)	2.626(2.006)	1.362(2.131)
Every month	-5.720(4.979)	-1.585(4.920)	-5.336(3.769)	7.264(9.460)	-3.863(3.570)	-2.730(3.941)
Once few months	4.755(7.444)	18.59**(6.786)	5.969(5.466)	35.25**(11.33)	11.07*(4.891)	9.897(5.262)
Observations	437	730	778	389	1,085	940

a. Results are presented as standardised regression coefficient (standard error), *p<0.05; **p<0.01; ***p<0.001.
